# A Finger-Stick Whole-Blood HIV Self-Test as an HIV Screening Tool Adapted to the General Public

**DOI:** 10.1371/journal.pone.0146755

**Published:** 2016-02-16

**Authors:** Thierry Prazuck, Stephen Karon, Camelia Gubavu, Jerome Andre, Jean Marie Legall, Elisabeth Bouvet, Georges Kreplak, Jean Paul Teglas, Gilles Pialoux

**Affiliations:** 1 Department of Infectious Diseases, Centre Hospitalier Régional, Orléans, France; 2 HF Prevention, Trappes, France; 3 Aides, Paris, France; 4 Department of Infectious Diseases, Hôpital Universitaire Bichat Claude Bernard, Paris, France; 5 Centre de Biologie du Chemin Vert (CBCV), Paris, France; 6 INSERM INED, U822, Hôpital Kremlin Bicêtre, Le Kremlin-Bicêtre, France; 7 Department of Infectious Diseases, Hôpital Tenon, Paris, France; University of Athens, Medical School, GREECE

## Abstract

**Background:**

In 2013, the French Health Authority approved the use of HIV self-tests in pharmacies for the general public. This screening tool will allow an increase in the number of screenings and a reduction in the delay between infection and diagnosis, thus reducing the risk of further infections. We previously compared 5 HIV-self test candidates (4 oral fluid and one whole blood) and demonstrated that the whole blood HIV test exhibited the optimal level of performance (sensitivity/specificity). We studied the practicability of an easy-to-use finger-stick whole blood HIV self-test “autotest VIH^®”^, when used in the general public.

**Methods and Materials:**

This multicenter cross-sectional study involved 411 participants from the Parisian region (AIDES and HF association) between April and July 2014 and was divided into 2 separate studies: one evaluating the capability of participants to obtain an interpretable result using only the information notice, and a second evaluating the interpretation of test results, using a provided chart.

**Results:**

A total of 411 consenting participants, 264 in the first study and 147 in the second, were included. All participants were over 18 years of age. In the first study, 99.2% of the 264 participants correctly administered the auto-test, and 21.2% needed, upon their request, telephone assistance. Ninety-two percent of participants responded that the test was easy/very easy to perform, and 93.5% did not find any difficulty obtaining a sufficient good quantity of blood. In the second study, 98.1% of the 147 participants correctly interpreted the results. The reading/interpretation errors concerned the negative (2.1%) or the indeterminate (3.3%) auto-tests.

**Conclusions:**

The success rate of handling and interpretation of this self-test is very satisfactory, demonstrating its potential for use by the general public and its utility to increase the number of opportunities to detect HIV patients.

## Introduction

In France, it is estimated that approximately 150,000 persons are HIV positive, among whom 30,000 do not know their seropositivity [1]. The annual National Public Health Institute (INVS) reports have shown that the number of new HIV cases per year is approximately 6,500, which has been stable since 2008 [2]. A significant number of these new cases are discovered at an advanced stage as a result of severe immunodeficiency [3]. In these patients, we observe an increased rate of mortality and morbidity, which negatively impacts quality of life. In Europe, 15%-38% of HIV-positive persons are diagnosed at late stages when the CD4 count is low and the blood viral load is high [2].

HIV self-tests have been in development since 1996 with the goal of reducing the number of HIV-infected persons [4]. However, despite their high sensitivity and specificity (>99.9%), the actual use of self-tests by the general public has never reached anticipated levels [5,6]. Newer and easier to perform tests have since been developed. Their main advantages are acceptability, confidentiality, accuracy after the three-month window period and accessibility [7,8]. The purpose of these self-tests is to minimize the number of HIV-infected persons who would not otherwise subject themselves to testing in healthcare facilities.

Rapid HIV tests using either peripheral blood samples or oral fluid have been available at healthcare centers and through AIDS associations since 2010, but only trained personnel were authorized to use them.

In 2012, the United States (U.S.) Food and Drug Administration (FDA) approved in-home HIV self-tests [9,10] that do not require any prior training, only the use of an information/instruction notice. Therefore, in 2013, the French Ministry of Social, Health and Women Rights and various expert committees (CNS: National AIDS Council) pronounced that they were in favor of the commercialization of HIV in-home tests to facilitate access to screening for the general public, with prior information and adequate support. The CNS estimates that the introduction of self-testing will help diagnose 4,000 HIV infections and prevent another 400 infections after the first year of commercialization [11].

In 2013, we conducted a study that compared the sensitivity of five HIV self-tests (one approved for in-home use, Oraquick^®^, and four point-of-care test candidates for in-home use), four oral fluid-based tests and one blood-based test. Based on this analysis, we concluded that the blood test is more sensitive than the oral fluid-based tests [12]. Given these results, we performed two studies mandated by French health authorities to evaluate the finger-stick whole-blood HIV home test from this study. We assessed the practicability of the test and the ability of study participants to interpret the test results. The participants had not been trained to perform the test; therefore, the testing conditions were similar to those during self-testing.

## Materials and Methods

The protocol was approved by the ethics committee at the Centre Hospitalier Regional d’Orleans, which includes physicians, biologists, pharmacists, statisticians and regulatory affairs officers, at the February 12, 2014 session. All participants gave their verbal informed consent to participate in this study. Written consent was not required because this study was a non-interventional study. Consent was collected anonymously on each evaluation questionnaire. This procedure, including the information letter, was previously submitted and approved by the ethics committee.

This multicenter cross-sectional study was performed between April 2014 and July 2014. The participants were selected during outreach screening programs conducted by the AIDES and HF-Prevention associations (i.e., at commercial centers and mobile screening units in urban centers) and during screening tests performed at the Center for Anonymous and Free HIV Testing (CDAG) at Bichat Claude Bernard University Hospital. The protocol was approved by the hospital local ethics committee.

In this study, we used the finger-stick whole-blood HIV test (“autotest VIH^®^,” AAZ-LMB, Rungis, France). This test uses a combination of a specific antibody binding protein that is conjugated to colloidal gold dye particles and HIV 1/2 antigens, which are bound to the solid phase membrane. The test procedure is explained in Table 1. The quantity of blood needed to perform the test is only 2.5 μl.

**Table 1:** Instructional notice.

Test procedure
1. Preparation
a. The participant must first open the pouch and remove all components. Then remove the buffer vial from the top of the sampler and put it in the holder.
2. Specimen application
a. Clean your finger with the wipe provided. Use the lancet needle to obtain drops of blood. The first drop of blood is wiped off with the compress. Next, the participant must press gently on the fingertip to obtain a second drop of blood that is placed in contact with the sampler tip until the tip is full.
b. Put the holder that contains the buffer vial on a flat surface.
c. Firmly press the sampler tip through the foil cover to the bottom of the vial until the Sampler and the buffer snap together. When properly seated, it will snap 3 times: snap 1 through foil, snap 2 into the cap, and snap 3 for the seat and seal.
d. Less than a minute after pressing the sampler into the buffer, a pink trail will appear on the sampler. If this pink trail does not appear, press hard to completely insert the sampler.
e. Wait 15 minutes and then read the results. Do not read the results if 20 or more minutes have passed.

The study was divided into 2 substudies: the first substudy evaluated the ability of participants to correctly perform the self-test, and the second substudy evaluated the ability of participants to read and correctly interpret the test results. Written, self-administered questionnaires were used to evaluate the participants’ opinions about the test and their level of satisfaction with the testing procedures.

### Selection of participants

All participants were volunteers recruited during screening programs conducted by the AIDES and HF-Prevention associations and at the Anonymous and Free Testing Center (Bichat Hospital, Paris). The inclusion criteria were an age ≥ 18 years, willingness to be tested for HIV, capacity to speak and understand French and willingness to give consent to participate in the study.

Overall, 411 participants were included in the study: 264 in the first substudy and 147 in the second substudy. In addition, 6 persons were excluded, including 4 minors and 2 adults who did not read the information notice.

### Substudy 1: Usage of the self-test

In a confidential room supervised by an observer, each participant received the kit that included the test, its accessories (disinfectant wipe, compress…) and information on how to use the kit. The observer confirmed the different steps that were followed by the participant on a standardized sheet.

Each participant had the option of performing the test alone or with assistance from the observer. This assistance was provided using the same procedure available by the hotline that was established with the commercialization of the test in France (September 15, 2015). Fifteen minutes later, the participant was invited to leave the room and to fill in the satisfaction questionnaire (Table 2). In this substudy, the participant was not allowed to read the results of their test because the device was not yet authorized to be used by a non-professional at the time. Next, the observer photographed the test to document the correct or incorrect usage of the test (if the test is performed correctly, a control line will appear). Finally, the participant moved to the next room with a trained staff member who performed the regular HIV rapid test, including pre- and post-counseling.

**Table 2:** Satisfaction questionnaire.

Satisfaction questionnaire
1. How would you evaluate the preparation of the kit components?
a. very easy
b. rather easy
c. rather difficult
d. very difficult
2. How would you evaluate the specimen application?
a. very easy
b. rather easy
c. rather difficult
d. very difficult
3. How would you evaluate the execution of the test?
a. very easy
b. rather easy
c. rather difficult
d. very difficult
4. How difficult was it to understand the instructions?
a. very easy
b. rather easy
c. rather difficult
d. very difficult

### Substudy 2: Reading and interpretation of the results

In a confidential room, each participant was shown a basket containing 6 standardized test results (2 positives, 2 negatives and 2 invalids). The participant had to randomly choose four out of the six standardized tests and write down the results for each test using a chart to interpret the test results. Among the 2 positives, one exhibited a plain line and the other a faint line. However, the positive line is usually lighter than the control line. At the end of the session, the participant was asked to fill in a satisfaction questionnaire.

### Statistical analysis

The data were analyzed using STATA/SE 13.1 (StataCorp LP, USA). The results were presented as a 95% confidence interval (CI) using the Clopper-Pearson method with independence of observations.

For the second substudy, the non-independence of observations for the overall positive tests was considered using a calculation method that included clustering of the participants and the inner-group correlations.

## Results

The demographic characteristics of the study population in each substudy are presented in Table 3.

**Table 3:** Demographic characteristics of the study population.

Characteristics	Substudy 1		Substudy 2	
	(N = 264)		(N = 147)	
	No.	%	No.	%
Age <30 years old	149	57	118	80
Male	158	60	66	45
MSM	87	33	13	9
Post-graduate	149	56	72	49
Never tested for HIV	44	17	44	30

**Substudy 1.** Validation of the ability of participants to obtain an interpretable result using the finger-stick whole-blood self-test and the information notice in a supervised setting.

**Fig 1:**
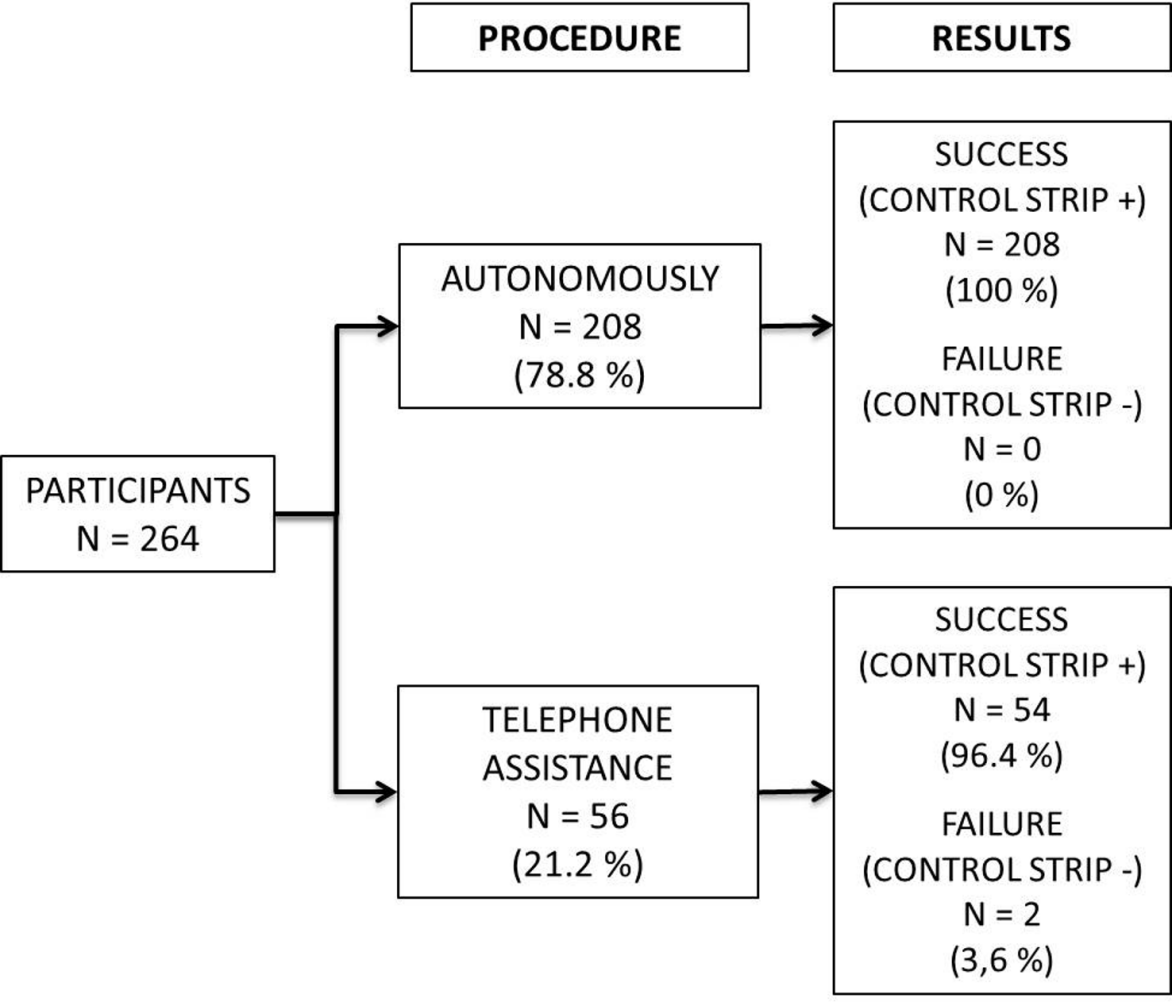
Ability of participants to obtain an interpretable result using the finger-stick whole-blood self-test and the information notice in a supervised setting.

A total of 264 participants were included in the first substudy, of whom 208 (78.8%) used the test by themselves and 56 (21.2%) needed assistance. Overall, 262 (99.2%; 95% CI 97.3–99.9) participants correctly used the self-test and succeeded in obtaining an interpretable result. All participants who performed the test autonomously and 54/56 (96.4%; 95% CI 87.7–99.6) of those who needed assistance successfully interpreted the test (Fig 1). Two participants failed to correctly use the test despite the use of technical support

Regarding the practicability of the self-test: 125 (47.3%) participants responded that the preparation was very easy, 119 (45.1%) responded that it was easy, 19 (7.2%) responded that it was rather difficult and 1 (0.4%) responded that it was very difficult. The 2 participants who failed to correctly use the test either did not answer this question or found that it was rather difficult. When asked about the difficulty of obtaining the blood specimen: 117 (44.3%) participants found it very easy, 131 (49.6%) found it rather easy, 12 (4.6%) found it rather difficult and 4 (1.5%) found it very difficult. In addition, 1 participant who found the test very difficult failed to correctly use the test.

A total of 134 (50.8%) participants responded that the test was very easy to perform, 107 (40.5%) found the test rather easy to perform, 20 (7.6%) found it rather difficult to perform and 2 (0.8%) found it very difficult. The participants who failed to correctly use the test did not answer this question. A total of 115 (43.6%) participants responded that the instruction notice was easy to interpret, 139 (52.6%) found it rather easy to interpret, 9 (3.4%) found it rather difficult to interpret and 4 (0.4%) found it very difficult.

**Substudy 2.** Validation of the ability of participants to read and interpret the HIV self-test results using a chart from a panel of 6 standardized tests.

**Fig 2:**
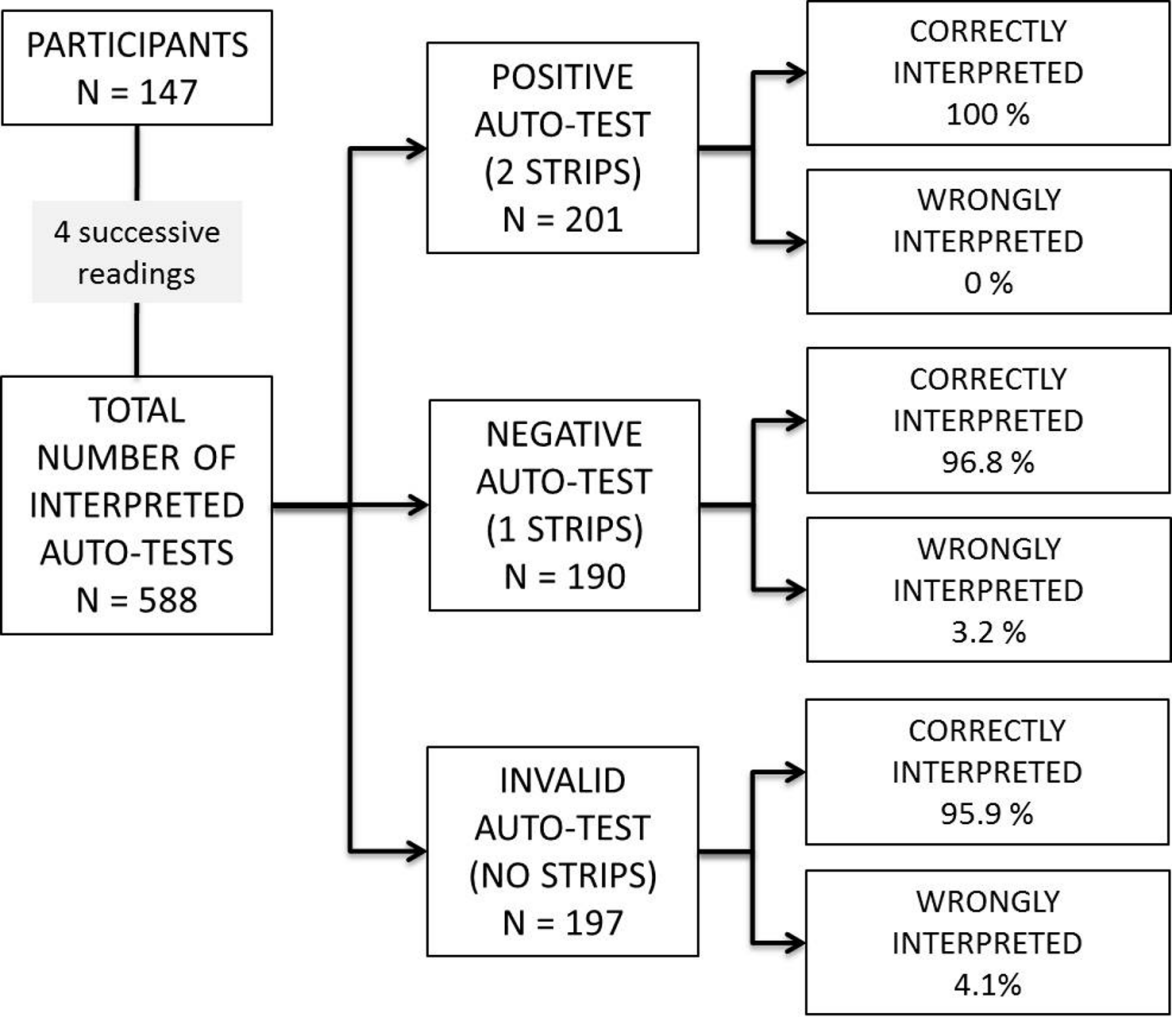
Ability of participants to read and interpret the HIV self-test results using a chart from a panel of 6 standardized tests.

A total of 588 standardized tests were interpreted by the 147 participants: 201 positive, 190 negative and 197 invalid. Eleven participants did not have to interpret a positive test (was not drawn), 8 of them had a negative result and 6 of them had an invalid result (Fig 2).

Overall, 571 (97.1%, 95% CI 95.9–99.1) tests were correctly interpreted, whereas 14 (2.9%) tests were misinterpreted. Six negative tests were misinterpreted: 4 as positive, and 2 as invalid; 8 invalid tests were falsely interpreted as positive (4) or negative (4).

A total of 142 (96.6%) participants responded that interpreting the positive tests was easy (72.6% very easy and 24% rather easy) compared with the invalid test (93.9%, most errors registered). Participants less than 30 years of age performed better than those who were older than 30 (75% vs. 57% with p<0.05).

## Discussion

Rapid HIV tests could potentially be sold over the counter for self-testing in France. These tests have been validated for use in the U.S. Several authors have proposed that self-testing can be an innovative component of community-wide HIV prevention strategies [13,14) by providing testing to persons who do not wish to reveal their sexual practices for reasons of stigma or confidentiality. Such persons could self-test when they perceive they are at risk of infection. Other authors have commented that if a particular diagnostic technology is adequate, it is difficult to justify restricting access to it [15].

Oral fluid-based HIV self-tests have been commercialized in the U.S. or evaluated in various African countries [8, 16, 17]. The popular Oraquick home test in the U.S. was shown to have a high positive predictive value (PPV) in oral specimens collected from high-prevalence settings. However, a slightly lower sensitivity and PPV has been found in oral specimens collected from individuals in low-prevalence settings, such as France, which must be taken into consideration before expanding this oral test worldwide [18].

In France, a low HIV prevalence country (a HIV prevalence of 0.3%), a finger-stick whole-blood HIV self-test has been proposed for self-testing instead of oral fluid-based self-testing. This whole-blood self-test has a sensitivity of 100% and uses a sample as small as 2.5 μl. The current study showed that requiring a whole blood sample does not limit use of the test by a non-trained population. For the general public, Figueroa et al [19] suggest that the main concern about HIV self-tests is not the type of test but the test’s accuracy in detecting HIV.

The first substudy showed that more than 99.2% of the participants (n = 264) who were not previously trained correctly used the finger-stick whole-blood self-test either autonomously or with assistance. Overall, 21.2% of participants needed technical support, which emphasizes its importance. Following the commercialization of this in-home test, a hotline was made available. Most participants found that the written and visual information was easy to read, interpret and execute. These findings validate the use of the self-test by the general public.

In the second substudy, we analyzed the capacity of participants to correctly interpret the results of 4 standardized tests. The majority (97.1%) of participants succeeded, but 2.9% of the participants made errors, mostly when reading an invalid test. In practice, every misinterpreted test leads to 3 possible consequences.

Interpreting a negative/invalid test as positive leads to further testing in healthcare centers. If a negative/positive test is interpreted as invalid, the test will be repeated, with or without assistance, as written in the information notice. If an invalid/positive test is read as negative, then the individual is at risk of being infected and could further spread the infection. This latter possibility reveals the limits of self-tests, and all individuals at a high risk of being infected are advised to seek assistance at a healthcare center. However, in our study, all positive tests were correctly interpreted, which is an encouraging result because we assume that persons reading a positive test will decide to seek appropriate care. Misinterpretation of invalid or negative results should lead to a second test, or calling the hotline for assistance.

The World Health Organization (WHO) identified the following key populations for HIV self-tests: men who have sex with men (MSM), sex workers (SWs), people who inject drugs (PWID), transgender people and people in prisons who are disproportionately affected by HIV [19]. However, HIV self-tests are sold to anyone without a medical prescription. It is therefore difficult to predict which populations will buy the HIV self-test and for which purpose they will use it in real life (i.e., recent HIV risk, first HIV testing, or reluctance to seek supervised HIV testing in a medical center).

A critical issue for HIV self-tests is the 3-month window period after infection. The instructions for use on the test packaging clearly alert persons that a very recent risk of HIV infection (within the last three months) may result in a false negative and an additional test should be performed after the 3-month window period. Persons with very recent HIV risk who do not read the instructions for use on the packaging could receive false reassurance that they are uninfected. However, assistance is available by phone for persons who are alone when they receive their results, which is a key issue to address before the commercialization of any HIV home test. Sida Info Service, a 24-hour phone assistance service that specializes in HIV in France, gives advice to individuals who take the self-test, including the quickest way to enter into care with specialists closest to the individual’s home.

The next step for persons who receive a positive test result is entering into care; however, it is difficult to predict when patients will seek care. Two studies [20,21] reported actual linkage and enrollment in care following HIV self-testing. Katz [20] reported two participants with reactive self-test results who were diagnosed as HIV positive: one participant searched immediately for additional HIV testing and care and the other participant waited two months before seeking further HIV testing and care. A study in low-income countries (LICs) reported that 50% of MSM would seek post-test counseling and confirmation of results and that 75% of FSWs stated they would go to health facilities for confirmation after self-testing for HIV [22]. In France, a large National Agency for AIDS Research (ANRS) cohort of MSM will be followed up to estimate the frequency of HIV self-test usage and the links to care when the tests are positive.

Because HIV home tests will be sold in private pharmacies, persons with positive tests may return to their pharmacists to seek advice. A large training program for pharmacists is ongoing in France to help them answer questions from patients and to facilitate patient access to specialized care.

A serious limitation of HIV self-tests may be the cost of the test. Willingness to pay for self-tests varies across populations, country income settings, type of specimen collection, and the type of approach. In high-income countries, a recent literature review found that study participants were willing to pay between US$20 and US$50 for HIV self-tests [19]. In France, the test is sold in pharmacies for US$22 to US$32.

Nevertheless, the risk/benefit ratio favors the commercialization of HIV self-tests.

In this study, the only socio-demographic characteristic that was associated with better performance at reading the tests was age (p<0.05), and persons with a relatively low education level correctly administered and interpreted the home test.

## Conclusion

These 2 substudies indicate that the finger-stick whole-blood HIV self-test “autotest VIH^®^” is practical and that users of the test correctly interpreted the results. Given these results, this test was made widely available in France on September 15, 2015.
